# Fluazinam

**DOI:** 10.1107/S1600536813023210

**Published:** 2013-08-23

**Authors:** Youngeun Jeon, Jineun Kim, Sangjin Lee, Tae Ho Kim

**Affiliations:** a Department of Chemistry and Research Institute of Natural Sciences, Gyeongsang National University, Jinju 660-701, Republic of Korea

## Abstract

In the asymmetric unit of the title compound {systematic name: 3-chloro-*N*-[3-chloro-5-(tri­fluoro­meth­yl)pyridin-2-yl]-2,6-di­nitro-4-(tri­fluoro­methyl)­aniline}, C_13_H_4_Cl_2_F_6_N_4_O_4_, which is the fungicide fluazinam, the dihedral angle between the pyridine and benzene ring planes is 42.20 (4)°. In the crystal, pairs of N—H⋯F hydrogen bonds link the mol­ecules into inversion dimers which are linked by C—Cl⋯π [Cl⋯ring centroid = 3.3618 (4) A °] and N—O⋯π [O⋯ring centroid = 3.1885 (16) Å] inter­actions into chains along [100]. In addition, short Cl⋯Cl, O⋯Cl, and F⋯F contacts [3.4676 (7), 3.2371 (13) and 2.7910 (15) Å] are present which connect the chains, yielding a three-dimensional network.

## Related literature

For information on the toxicity and fungicidal properties of the title compound, see: Yoshida & Yukimoto (1993 ▶); Draper *et al.* (2003 ▶). For a related structure, see: McCullough *et al.* (1972 ▶).
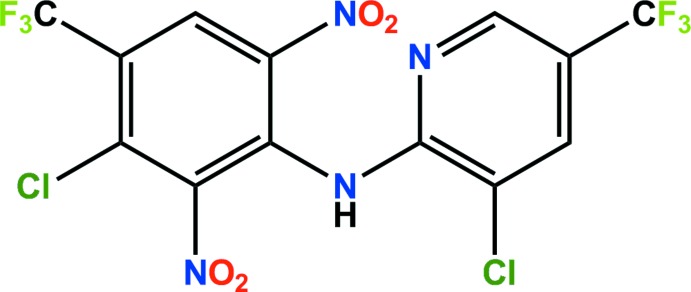


## Experimental

### 

#### Crystal data

C_13_H_4_Cl_2_F_6_N_4_O_4_
*M* *_r_* = 465.10
Triclinic, 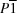

*a* = 8.9546 (1) Å
*b* = 9.0724 (1) Å
*c* = 10.6818 (2) Å
α = 79.556 (1)°
β = 75.420 (1)°
γ = 83.451 (1)°
*V* = 823.79 (2) Å^3^
*Z* = 2
Mo *K*α radiation
μ = 0.49 mm^−1^
*T* = 173 K
0.30 × 0.18 × 0.15 mm

#### Data collection

Bruker APEXII CCD detector diffractometer
Absorption correction: multi-scan (*SADABS*; Bruker, 2006 ▶) *T*_min_ = 0.867, *T*_max_ = 0.930
15193 measured reflections
4096 independent reflections
3662 reflections with *I* > 2σ(*I*)
*R*_int_ = 0.022

#### Refinement

*R*[*F* ^2^ > 2σ(*F* ^2^)] = 0.032
*wR*(*F* ^2^) = 0.085
*S* = 1.04
4096 reflections
262 parameters
H-atom parameters constrained
Δρ_max_ = 0.47 e Å^−3^
Δρ_min_ = −0.45 e Å^−3^

### 

Data collection: *APEX2* (Bruker, 2006 ▶); cell refinement: *SAINT* (Bruker, 2006 ▶); data reduction: *SAINT*; program(s) used to solve structure: *SHELXS97* (Sheldrick, 2008 ▶); program(s) used to refine structure: *SHELXL97* (Sheldrick, 2008 ▶); molecular graphics: *SHELXTL* (Sheldrick, 2008 ▶) and *DIAMOND* (Brandenburg, 1998 ▶); software used to prepare material for publication: *SHELXTL*.

## Supplementary Material

Crystal structure: contains datablock(s) global, I. DOI: 10.1107/S1600536813023210/kj2230sup1.cif

Structure factors: contains datablock(s) I. DOI: 10.1107/S1600536813023210/kj2230Isup2.hkl

Supplementary material file. DOI: 10.1107/S1600536813023210/kj2230Isup3.cml

Additional supplementary materials:  crystallographic information; 3D view; checkCIF report

## Figures and Tables

**Table 1:** Hydrogen-bond geometry (Å, °)

*D*—H⋯*A*	*D*—H	H⋯*A*	*D*⋯*A*	*D*—H⋯*A*
N3—H3⋯F3^i^	0.88	2.52	3.0690 (15)	121

Symmetry code: (i) 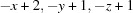.

**Fig. 1.**
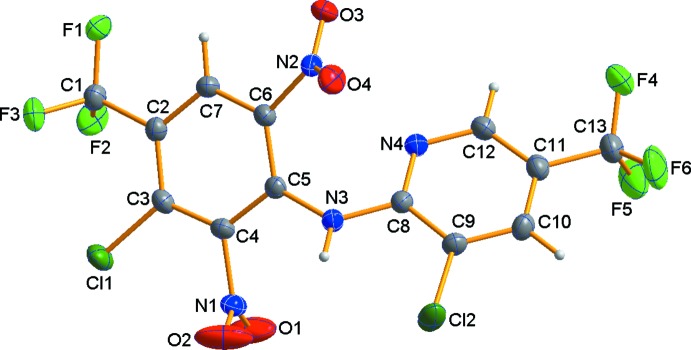
The molecular structure of the title compound. Displacement ellipsoids are drawn at the 50% probability level. H atoms are shown as small spheres of arbitrary radius.

**Fig. 2.**
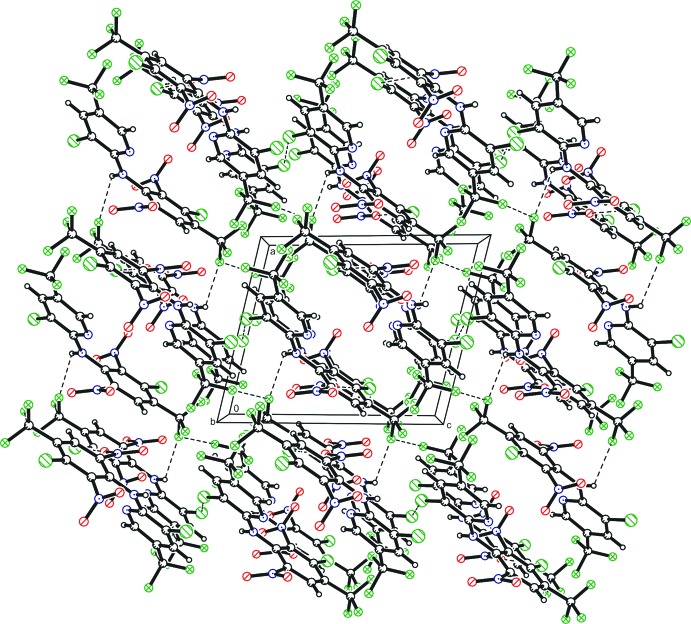
Crystal packing of the title compound with N—H···F hydrogen bonds and weak intermolecular Cl···Cl and F···F interactions shown as dashed lines.

## supplementary crystallographic information

### 1. Comment

The title compound fluazinam, C_13_H_4_Cl_2_F_6_N_4_O_4_, is a broad spectrum contact fungicide that can be applied as a foliar spray or soil treatment (Yoshida *et al.*, 1993; Draper *et al.*, 2003) and its crystal structure is reported herein. In this compound (Fig. 1), the dihedral angle between the pyridyl ring and the phenyl ring is 42.20 (4)°. All bond lengths and bond angles are normal and comparable to those observed in the crystal structures of a similar compound (McCullough *et al.*, 1972). In the crystal structure (Fig. 2), an intermolecular N—H···F hydrogen bond is observed (Table 1), giving a dimer structure. In this structure there are both a C3—Cl1···π interaction with the pyridyl ring [Cl1···*Cg*1^ii^ = 3.3618 (4) Å] and a N1—O1···π interaction with the phenyl ring [O1···*Cg*2^ii^ = 3.1885 (16) Å]. In addition, short Cl···Cl, O···Cl, and F···F contacts [Cl2···Cl2^iii^, 3.4676 (7) Å, O3···Cl1^iv^, 3.2371 (13) Å, and F3···F6^v^, 2.7910 (15) Å] are present [for symmetry codes: (ii), *-x*+1, *-y*+1, *-z*+1, (iii), *-x*+1, *-y*+1, *-z*+2, (iv), *x*, *y* - 1, *z*, and (v), *x* + 1, *y* + 1, *z* + 1]. A three-dimensional network is formed by the hydrogen bond and these interactions.

### 2. Experimental

The title compound was purchased from the Dr. Ehrenstorfer GmbH Company. Slow evaporation of a solution in CH_2_Cl_2_ gave single crystals suitable for X-ray analysis.

### 3. Refinement

All H-atoms were positioned geometrically and refined using a riding model with d(N—H) = 0.88 Å, *U*_iso_ = 1.2*U*_eq_(C) for amine and d(C—H) = 0.95 Å, *U*_iso_ = 1.2*U*_eq_(C) for C*sp*^2^—H.

### Crystal data

**Table:**

C_13_H_4_Cl_2_F_6_N_4_O_4_	*Z* = 2
*M**_r_* = 465.10	*F*(000) = 460
Triclinic, *P*1	*D*_x_ = 1.875 Mg m^−^^3^
Hall symbol: -P 1	Mo *K*α radiation, λ = 0.71073 Å
*a* = 8.9546 (1) Å	Cell parameters from 8261 reflections
*b* = 9.0724 (1) Å	θ = 2.4–28.3°
*c* = 10.6818 (2) Å	µ = 0.49 mm^−^^1^
α = 79.556 (1)°	*T* = 173 K
β = 75.420 (1)°	Block, yellow
γ = 83.451 (1)°	0.30 × 0.18 × 0.15 mm
*V* = 823.79 (2) Å^3^	

### Data collection

**Table:**

Bruker APEXII CCD detector diffractometer	4096 independent reflections
Radiation source: fine-focus sealed tube	3662 reflections with *I* > 2σ(*I*)
Graphite monochromator	*R*_int_ = 0.022
φ and ω scans	θ_max_ = 28.4°, θ_min_ = 2.0°
Absorption correction: multi-scan (*SADABS*; Bruker, 2006)	*h* = −11→11
*T*_min_ = 0.867, *T*_max_ = 0.930	*k* = −12→10
15193 measured reflections	*l* = −14→14

### Refinement

**Table:**

Refinement on *F*^2^	Primary atom site location: structure-invariant direct methods
Least-squares matrix: full	Secondary atom site location: difference Fourier map
*R*[*F*^2^ > 2σ(*F*^2^)] = 0.032	Hydrogen site location: inferred from neighbouring sites
*wR*(*F*^2^) = 0.085	H-atom parameters constrained
*S* = 1.04	*w* = 1/[σ^2^(*F*_o_^2^) + (0.0402*P*)^2^ + 0.3959*P*] where *P* = (*F*_o_^2^ + 2*F*_c_^2^)/3
4096 reflections	(Δ/σ)_max_ < 0.001
262 parameters	Δρ_max_ = 0.47 e Å^−^^3^
0 restraints	Δρ_min_ = −0.45 e Å^−^^3^

### Special details

**Table:**

Geometry. All e.s.d.'s (except the e.s.d. in the dihedral angle between two l.s. planes) are estimated using the full covariance matrix. The cell e.s.d.'s are taken into account individually in the estimation of e.s.d.'s in distances, angles and torsion angles; correlations between e.s.d.'s in cell parameters are only used when they are defined by crystal symmetry. An approximate (isotropic) treatment of cell e.s.d.'s is used for estimating e.s.d.'s involving l.s. planes.
Refinement. Refinement of *F*^2^ against ALL reflections. The weighted *R*-factor *wR* and goodness of fit *S* are based on *F*^2^, conventional *R*-factors *R* are based on *F*, with *F* set to zero for negative *F*^2^. The threshold expression of *F*^2^ > σ(*F*^2^) is used only for calculating *R*-factors(gt) *etc*. and is not relevant to the choice of reflections for refinement. *R*-factors based on *F*^2^ are statistically about twice as large as those based on *F*, and *R*- factors based on ALL data will be even larger.

### Fractional atomic coordinates and isotropic or equivalent isotropic displacement parameters (Å^2^)

**Table:**

	*x*	*y*	*z*	*U*_iso_*/*U*_eq_	
Cl1	0.83432 (5)	0.72367 (4)	0.34806 (4)	0.03069 (10)	
Cl2	0.43809 (5)	0.32745 (5)	0.99480 (4)	0.03760 (11)	
F1	1.06852 (11)	0.34123 (11)	0.15585 (9)	0.0342 (2)	
F2	0.90981 (11)	0.53398 (12)	0.12664 (9)	0.0369 (2)	
F3	1.11164 (11)	0.55470 (11)	0.19484 (10)	0.0357 (2)	
F4	0.25749 (13)	−0.25110 (12)	0.84106 (12)	0.0484 (3)	
F5	0.07528 (12)	−0.07681 (13)	0.86228 (14)	0.0542 (3)	
F6	0.17049 (16)	−0.17401 (14)	1.02625 (11)	0.0572 (3)	
N1	0.63148 (15)	0.61200 (13)	0.60382 (12)	0.0257 (3)	
N2	0.82927 (13)	0.07922 (13)	0.58834 (12)	0.0239 (2)	
N3	0.62868 (14)	0.31709 (14)	0.72658 (11)	0.0234 (2)	
H3	0.6329	0.3764	0.7821	0.028*	
N4	0.52522 (14)	0.10739 (14)	0.69279 (12)	0.0240 (2)	
O1	0.51649 (16)	0.65800 (18)	0.56501 (17)	0.0595 (4)	
O2	0.6693 (2)	0.65407 (19)	0.68990 (16)	0.0653 (5)	
O3	0.83309 (13)	−0.01445 (12)	0.51781 (11)	0.0321 (2)	
O4	0.84483 (13)	0.05111 (13)	0.70083 (11)	0.0321 (2)	
C1	1.00024 (17)	0.46373 (17)	0.20469 (14)	0.0257 (3)	
C2	0.90782 (15)	0.42479 (15)	0.34387 (13)	0.0210 (3)	
C3	0.82543 (16)	0.53636 (15)	0.41309 (13)	0.0210 (3)	
C4	0.73336 (15)	0.49449 (15)	0.53729 (13)	0.0209 (3)	
C5	0.72421 (15)	0.34551 (15)	0.60148 (13)	0.0197 (3)	
C6	0.81363 (15)	0.23826 (15)	0.53006 (13)	0.0204 (3)	
C7	0.90021 (16)	0.27574 (15)	0.40317 (13)	0.0215 (3)	
H7	0.9548	0.1987	0.3564	0.026*	
C8	0.52749 (15)	0.20410 (15)	0.77180 (13)	0.0208 (3)	
C9	0.42873 (16)	0.19730 (16)	0.89770 (14)	0.0238 (3)	
C10	0.32506 (17)	0.08814 (17)	0.94103 (14)	0.0267 (3)	
H10	0.2564	0.0818	1.0253	0.032*	
C11	0.32343 (17)	−0.01298 (16)	0.85797 (15)	0.0257 (3)	
C12	0.42492 (17)	0.00019 (16)	0.73578 (15)	0.0261 (3)	
H12	0.4234	−0.0698	0.6800	0.031*	
C13	0.20746 (19)	−0.12907 (18)	0.89734 (17)	0.0332 (3)	

### Atomic displacement parameters (Å^2^)

**Table:**

	*U*^11^	*U*^22^	*U*^33^	*U*^12^	*U*^13^	*U*^23^
Cl1	0.0402 (2)	0.01712 (16)	0.03235 (19)	−0.00298 (13)	−0.00704 (15)	0.00063 (13)
Cl2	0.0392 (2)	0.0455 (2)	0.02904 (19)	−0.01359 (17)	0.00518 (15)	−0.02051 (17)
F1	0.0373 (5)	0.0320 (5)	0.0270 (4)	−0.0033 (4)	0.0067 (4)	−0.0076 (4)
F2	0.0418 (5)	0.0452 (6)	0.0211 (4)	0.0003 (4)	−0.0090 (4)	0.0013 (4)
F3	0.0328 (5)	0.0331 (5)	0.0372 (5)	−0.0146 (4)	−0.0007 (4)	0.0018 (4)
F4	0.0441 (6)	0.0303 (5)	0.0704 (8)	−0.0134 (4)	−0.0029 (5)	−0.0154 (5)
F5	0.0303 (5)	0.0468 (7)	0.0869 (9)	−0.0104 (5)	−0.0176 (6)	−0.0038 (6)
F6	0.0738 (8)	0.0510 (7)	0.0409 (6)	−0.0357 (6)	0.0012 (6)	0.0069 (5)
N1	0.0302 (6)	0.0191 (6)	0.0264 (6)	0.0011 (5)	−0.0048 (5)	−0.0047 (5)
N2	0.0218 (6)	0.0194 (6)	0.0264 (6)	−0.0010 (4)	0.0003 (4)	−0.0018 (5)
N3	0.0264 (6)	0.0231 (6)	0.0203 (5)	−0.0068 (4)	0.0004 (4)	−0.0074 (5)
N4	0.0246 (6)	0.0230 (6)	0.0237 (6)	−0.0033 (4)	−0.0018 (4)	−0.0067 (5)
O1	0.0400 (7)	0.0658 (10)	0.0874 (11)	0.0247 (7)	−0.0296 (8)	−0.0471 (9)
O2	0.0887 (12)	0.0629 (10)	0.0628 (10)	0.0380 (9)	−0.0454 (9)	−0.0464 (8)
O3	0.0366 (6)	0.0195 (5)	0.0370 (6)	−0.0018 (4)	0.0002 (5)	−0.0092 (4)
O4	0.0354 (6)	0.0287 (6)	0.0283 (6)	0.0002 (4)	−0.0075 (4)	0.0039 (4)
C1	0.0263 (7)	0.0261 (7)	0.0224 (7)	−0.0049 (5)	−0.0024 (5)	−0.0013 (5)
C2	0.0211 (6)	0.0221 (6)	0.0195 (6)	−0.0036 (5)	−0.0044 (5)	−0.0021 (5)
C3	0.0245 (6)	0.0168 (6)	0.0227 (6)	−0.0032 (5)	−0.0081 (5)	−0.0009 (5)
C4	0.0232 (6)	0.0184 (6)	0.0223 (6)	−0.0007 (5)	−0.0057 (5)	−0.0064 (5)
C5	0.0199 (6)	0.0205 (6)	0.0191 (6)	−0.0034 (5)	−0.0040 (5)	−0.0044 (5)
C6	0.0213 (6)	0.0175 (6)	0.0218 (6)	−0.0019 (5)	−0.0042 (5)	−0.0025 (5)
C7	0.0225 (6)	0.0199 (6)	0.0218 (6)	−0.0011 (5)	−0.0032 (5)	−0.0052 (5)
C8	0.0205 (6)	0.0200 (6)	0.0209 (6)	−0.0011 (5)	−0.0033 (5)	−0.0031 (5)
C9	0.0254 (7)	0.0250 (7)	0.0209 (6)	−0.0016 (5)	−0.0027 (5)	−0.0072 (5)
C10	0.0248 (7)	0.0299 (8)	0.0220 (7)	−0.0044 (6)	0.0000 (5)	−0.0020 (6)
C11	0.0246 (7)	0.0215 (7)	0.0296 (7)	−0.0035 (5)	−0.0054 (5)	−0.0005 (6)
C12	0.0268 (7)	0.0219 (7)	0.0298 (7)	−0.0030 (5)	−0.0046 (6)	−0.0070 (6)
C13	0.0321 (8)	0.0272 (8)	0.0377 (8)	−0.0090 (6)	−0.0032 (6)	−0.0014 (6)

### Geometric parameters (Å, º)

**Table:**

Cl1—C3	1.7180 (13)	N4—C8	1.3280 (18)
Cl2—C9	1.7283 (14)	N4—C12	1.3391 (18)
F1—C1	1.3326 (17)	C1—C2	1.5090 (19)
F2—C1	1.3366 (17)	C2—C7	1.3868 (19)
F3—C1	1.3382 (17)	C2—C3	1.3936 (19)
F4—C13	1.334 (2)	C3—C4	1.3864 (19)
F5—C13	1.340 (2)	C4—C5	1.4017 (19)
F6—C13	1.330 (2)	C5—C6	1.3978 (18)
N1—O2	1.1911 (18)	C6—C7	1.3849 (18)
N1—O1	1.2084 (18)	C7—H7	0.9500
N1—C4	1.4834 (17)	C8—C9	1.4065 (19)
N2—O4	1.2220 (16)	C9—C10	1.373 (2)
N2—O3	1.2263 (16)	C10—C11	1.391 (2)
N2—C6	1.4701 (17)	C10—H10	0.9500
N3—C8	1.3846 (17)	C11—C12	1.384 (2)
N3—C5	1.3906 (17)	C11—C13	1.495 (2)
N3—H3	0.8800	C12—H12	0.9500
			
O2—N1—O1	125.30 (14)	C7—C6—C5	122.46 (12)
O2—N1—C4	117.99 (13)	C7—C6—N2	115.65 (12)
O1—N1—C4	116.71 (12)	C5—C6—N2	121.78 (12)
O4—N2—O3	125.24 (12)	C6—C7—C2	120.52 (13)
O4—N2—C6	117.51 (12)	C6—C7—H7	119.7
O3—N2—C6	117.15 (12)	C2—C7—H7	119.7
C8—N3—C5	126.00 (12)	N4—C8—N3	118.35 (12)
C8—N3—H3	117.0	N4—C8—C9	122.28 (13)
C5—N3—H3	117.0	N3—C8—C9	119.35 (12)
C8—N4—C12	118.20 (12)	C10—C9—C8	119.38 (13)
F1—C1—F2	107.50 (12)	C10—C9—Cl2	120.99 (11)
F1—C1—F3	107.14 (12)	C8—C9—Cl2	119.63 (11)
F2—C1—F3	106.99 (12)	C9—C10—C11	118.07 (13)
F1—C1—C2	111.51 (12)	C9—C10—H10	121.0
F2—C1—C2	111.11 (12)	C11—C10—H10	121.0
F3—C1—C2	112.33 (12)	C12—C11—C10	119.23 (13)
C7—C2—C3	119.09 (12)	C12—C11—C13	120.38 (14)
C7—C2—C1	119.92 (12)	C10—C11—C13	120.30 (14)
C3—C2—C1	120.96 (12)	N4—C12—C11	122.83 (14)
C4—C3—C2	118.91 (12)	N4—C12—H12	118.6
C4—C3—Cl1	119.39 (11)	C11—C12—H12	118.6
C2—C3—Cl1	121.70 (10)	F6—C13—F4	107.38 (14)
C3—C4—C5	123.74 (12)	F6—C13—F5	106.72 (14)
C3—C4—N1	118.60 (12)	F4—C13—F5	106.11 (14)
C5—C4—N1	117.56 (12)	F6—C13—C11	112.20 (14)
N3—C5—C6	126.22 (12)	F4—C13—C11	112.41 (13)
N3—C5—C4	118.65 (12)	F5—C13—C11	111.63 (13)
C6—C5—C4	115.13 (12)		
			
F1—C1—C2—C7	−1.24 (18)	O3—N2—C6—C7	−41.24 (17)
F2—C1—C2—C7	118.67 (14)	O4—N2—C6—C5	−40.98 (18)
F3—C1—C2—C7	−121.52 (14)	O3—N2—C6—C5	142.43 (13)
F1—C1—C2—C3	−179.22 (12)	C5—C6—C7—C2	3.5 (2)
F2—C1—C2—C3	−59.31 (17)	N2—C6—C7—C2	−172.80 (12)
F3—C1—C2—C3	60.50 (17)	C3—C2—C7—C6	−0.9 (2)
C7—C2—C3—C4	−2.56 (19)	C1—C2—C7—C6	−178.88 (12)
C1—C2—C3—C4	175.44 (12)	C12—N4—C8—N3	−178.61 (13)
C7—C2—C3—Cl1	177.84 (10)	C12—N4—C8—C9	−0.1 (2)
C1—C2—C3—Cl1	−4.16 (18)	C5—N3—C8—N4	3.9 (2)
C2—C3—C4—C5	3.7 (2)	C5—N3—C8—C9	−174.67 (13)
Cl1—C3—C4—C5	−176.73 (10)	N4—C8—C9—C10	−0.5 (2)
C2—C3—C4—N1	−172.68 (12)	N3—C8—C9—C10	178.00 (13)
Cl1—C3—C4—N1	6.93 (17)	N4—C8—C9—Cl2	−179.84 (11)
O2—N1—C4—C3	−104.70 (18)	N3—C8—C9—Cl2	−1.30 (19)
O1—N1—C4—C3	74.84 (19)	C8—C9—C10—C11	0.6 (2)
O2—N1—C4—C5	78.73 (19)	Cl2—C9—C10—C11	179.93 (11)
O1—N1—C4—C5	−101.72 (17)	C9—C10—C11—C12	−0.2 (2)
C8—N3—C5—C6	−43.9 (2)	C9—C10—C11—C13	−176.82 (14)
C8—N3—C5—C4	135.41 (14)	C8—N4—C12—C11	0.5 (2)
C3—C4—C5—N3	179.48 (12)	C10—C11—C12—N4	−0.4 (2)
N1—C4—C5—N3	−4.14 (18)	C13—C11—C12—N4	176.21 (14)
C3—C4—C5—C6	−1.2 (2)	C12—C11—C13—F6	151.47 (15)
N1—C4—C5—C6	175.22 (12)	C10—C11—C13—F6	−31.9 (2)
N3—C5—C6—C7	176.86 (13)	C12—C11—C13—F4	30.3 (2)
C4—C5—C6—C7	−2.45 (19)	C10—C11—C13—F4	−153.10 (14)
N3—C5—C6—N2	−7.1 (2)	C12—C11—C13—F5	−88.78 (18)
C4—C5—C6—N2	173.63 (12)	C10—C11—C13—F5	87.80 (19)
O4—N2—C6—C7	135.34 (13)		

### Hydrogen-bond geometry (Å, º)

**Table:**

*D*—H···*A*	*D*—H	H···*A*	*D*···*A*	*D*—H···*A*
N3—H3···F3^i^	0.88	2.52	3.0690 (15)	121

Symmetry code: (i) −*x*+2, −*y*+1, −*z*+1.
